# Avoidable mortality from respiratory tract infection and sudden unexplained death in children with chronic conditions: a data linkage study

**DOI:** 10.1136/archdischild-2017-314098

**Published:** 2018-07-14

**Authors:** Maximiliane L Verfürden, Ruth Gilbert, Neil Sebire, Pia Hardelid

**Affiliations:** 1 Population, Policy and Practice, UCL Great Ormond Street Institute of Child Health, London, UK; 4 Developmental Biology and Cancer Programme, UCL Great Ormond Street Institute of Child Health, London, UK

**Keywords:** avoidable mortality, chronic conditions, administrative data, respiratory mortality, sudden unexplained death

## Abstract

**Objective:**

To determine the risk of death from two potentially avoidable causes with different aetiologies: respiratory tract infection (RTI) and sudden unexplained death (SUD) in children with and without chronic conditions.

**Design:**

Whole-country, birth cohort study using linked administrative health databases from Scotland.

**Setting and participants:**

Children aged 2 months to less than 5 years in Scotland between 2000 and 2014.

**Main outcome measures:**

Relative risk of death (expressed as the HR) related to RTIs or SUD, in children with and without chronic conditions. We separately analysed deaths at ages 2–11 months and at 1–4 years and adjusted for birth characteristics, socioeconomic status and vaccination uptake using Cox regression.

**Results:**

The cohort comprised 761 172 children. Chronic conditions were recorded in 9.6% (n=72 901) of live births, 82.4% (n=173) of RTI-related deaths and 17.4% (n=49) of SUDs. Chronic conditions were very strongly associated with RTI mortality (2–11 months: HR 68.48, 95% CI (40.57 to 115.60), 1–4 years: HR 38.32, 95% CI (23.26 to 63.14)) and strongly associated with SUD (2–11 months: HR 2.42, 95% CI (1.67 to 3.63), 1–4 years: HR 2.53, 95% CI (1.36 to 4.71)).

**Conclusions:**

The very strong association with chronic conditions suggests that RTI-related mortality may sometimes be a consequence of a terminal decline and not possible to defer or prevent in all cases. Recording whether death was expected on death certificates could indicate which RTI-related deaths might be avoidable through healthcare and public health measures.

**What is already known on this topic?**

- Avoidable child mortality is used as an indicator for health system performance.
- Respiratory tract infection (RTI)-related mortality and sudden unexplained death are two of the most frequent causes of death in early childhood.

**What this study adds?**

- Children with chronic conditions were at substantially increased risk of RTI-related death and of sudden unexplained death.
- The majority of RTI-related deaths were in children with chronic conditions, some of these are likely to have been expected and were therefore potentially unavoidable.
- The introduction of an indicator of whether death was expected would allow a more precise quantification of potentially avoidable child mortality.

## Introduction

Chronic conditions account for more than 80% of non-injury deaths between 1 month and 5 years of age.^1 2^ The prevalence and complexity of chronic conditions in children is rising, as survival beyond the neonatal period improves for children born extremely prematurely.^3 4^ In developed countries, 13%–27% of all children have been diagnosed with a chronic condition, depending on the definition used.^4^

Child mortality is used as a benchmark for improvements in healthcare. However, the extent to which mortality among children with chronic conditions can be avoided by improved healthcare and prevention is not clear, particularly among children with multiple and life-limiting complex conditions.^5–7^

In this study, we focus on two outcomes: deaths related to respiratory tract infections (RTI) and sudden unexplained deaths (SUD). In industrialised countries they represent two of the most common causes of mortality in early childhood. RTI-related deaths and SUDs are potentially avoidable but are likely to have different aetiologies.^8^ To our knowledge, no cohort study has previously determined the association of underlying chronic conditions with these two causes of death in children.

RTI-related deaths may be prevented or delayed through timely vaccination and early recognition of infection and antibiotic treatment.^9^ However, severe RTIs may also be a consequence of terminal events in an expected death due to a child’s life-limiting chronic condition. A UK data linkage study found that 90% of children aged 1 month to 4 years who died with an RTI had at least one chronic condition.^1 2 9 10^ SUDs may be preventable through promoting safe sleeping, breast feeding, avoidance of maternal smoking and timely response to infection.^11 12^

According to official mortality statistics, RTIs or SUDs are recorded as underlying causes of death in 33% of all postneonatal deaths and in 12% of all deaths in children aged 1–5 years in the UK.^8 12 13^ These figures underestimate the contribution of RTIs, in particular as they exclude records of RTI as a contributory but not the main underlying cause of death.^9^

To determine whether RTI deaths or SUDs are useful for monitoring avoidable mortality, it is necessary to assess their association with chronic conditions.^6 7 14 15^ If a high proportion of deaths from these causes occur among children with chronic conditions, this may suggest that these deaths were unavoidable by treatment near to the time of death (but could be addressed by reducing upstream causes of chronic conditions).

We used linked, national hospitalisation and death registration data for Scotland to determine associations between chronic conditions and RTI mortality or SUD in live-born children aged 2 months to 5 years. Our findings aim to inform researchers and policymakers about the use of indicators for avoidable mortality in young children.

## Methods

### Study population

We derived a national electronic birth cohort of all children born in Scotland to Scottish resident mothers between October 1999 and December 2013. Births were identified using National Records for Scotland birth certificates and were linked to maternity, neonatal and administrative hospital records, vaccination uptake during infancy, outmigration and death certificates.^16^ Data sets were linked by the electronic Data Research and Innovation Service^17^ using deterministic linkage on the Community Health Index number, a unique identifier in the Scottish National Health Service.

Children were followed from 2 months to 4 completed years of age, death, outmigration or 31 December 2014, whichever occurred first. We excluded children who died before 2 months old since the vast majority of these deaths are associated with maternal health during pregnancy and delivery, preterm birth, intrapartum events and congenital anomalies, and therefore may not be avoidable through improved care after postnatal discharge.^8^ We excluded children weighing less than 500 g at birth, or born at less than 24 weeks’ gestation, to minimise erroneous inclusion of fetal deaths. We also excluded multiple births, since data from these children are prone to linkage error.

### Outcomes

To minimise underestimation of RTI-related mortality,^2 18^ we classified a death as RTI related if the following International Classification of Diseases (ICD)-10 codes were recorded in any of the contributory causes mentioned on the death registration or in a hospital admission within 30 days before death: A15, A16, A19 (tuberculosis of the respiratory system, miliary tuberculosis), A37 (whooping cough), B97.4 (respiratory syncytial virus), J00–J06 (acute upper respiratory infections), J09–J18 (influenza and pneumonia) and J20–J22 (other acute lower respiratory infections).

A death was classified as SUD if there was any record of the following ICD-10 codes on the death registration: R95 (sudden infant death syndrome), R96 (other sudden death, cause unknown), R98 (unattended death) and R99 (other ill-defined and unspecified causes of mortality). We focused on sudden *unexplained* death, which excludes unexpected but explained deaths such as W75 (accidental suffocation and strangulation in bed).

From these outcomes we excluded all deaths, where the underlying cause of death was an external cause (ICD-10 chapters: V–Y). Any death that according to our definition could be classified as both RTI and SUD was classified as SUD.

### Chronic conditions

We used a previously reported classification of chronic condition, defined by ICD-10 codes recorded on the death certificate or at previous hospital admissions. It reflects health conditions that are expected to require healthcare follow-up more than 12 months later in more than 50% of cases (online supplementary table 3).^2 19^ Children were classified as having a chronic condition if they had a relevant ICD-10 code recorded between birth and 11 completed months for analyses of deaths between 2 and 11 months and before 5 years for deaths in children aged 1–4 completed years. Children without a relevant ICD-10 code recorded were assumed to have no chronic condition.

10.1136/archdischild-2017-314098.supp1 Supplementary file 1

### Covariates

Birth characteristics included the baby’s sex and gestational age (coded into <33 weeks; 33–36 weeks; ≥37 weeks in the younger age group; and <37 and ≥37 weeks in the older age group due to small numbers).^20^ Socioeconomic status (SES) was measured by the Carstairs Index, a postcode sector indicator of material disadvantage^21^ coded in quintiles, and a binary indicator of teenage motherhood (mother was aged <20 years at delivery) as a proxy measure for individual-level SES, since teenage motherhood is strongly associated with material deprivation in the UK.^22^ We included vaccine delay as an indicator of engagement with preventive health services for deaths between 1 and 4 years to ensure all children could be exposed or not regardless of outcome. We defined delayed vaccine uptake during infancy by any vaccine either missing completely or given 3 or more months after the recommended age during infancy: *Haemophilus influenzae* type b (recommended at 2, 3 and 4 months of age), pneumococcal vaccine (2 and 4 months) or pertussis (2, 3 and 4 months).

We assumed that absence of a record indicating postmortem on the hospital discharge or death registration^23^ reflected an expected death or a data entry error.

### Statistical analyses

We separately analysed RTI-related mortality and SUD in children aged 2–11 months and 1–4 years to allow for expected differences in risk factors. We compared differences in characteristics between children with and without chronic conditions using χ^2^ tests and Fisher’s exact tests for expected cell sizes <5. A p value <0.05 was considered statistically significant.

We estimated RTI-related mortality and SUD rates per 100 000 child-years with 95% CIs according to each risk factor. We tested the assumptions of the Cox model and then fitted multivariable proportional hazard Cox regression models to the data to calculate adjusted risks of mortality related to RTI and SUD. Risk factors were added to the model in a forward, stepwise manner, determined a priori. We first included chronic conditions, then added birth characteristics, and teenage motherhood and deprivation quintile to explore the additional effects of SES not explained by chronic conditions. Lastly, we added delayed vaccine uptake (for children aged 1–4 years).

We imputed missing values (table 1) for gestational age and maternal age using chained multiple imputation, creating 15 imputed data sets.^24^ All statistical analyses were performed using Stata V.13.0.

**Table 1:** Characteristics of children aged 2 months to 4 years in Scotland between 2000 and 2014, by outcome and chronic condition status, stratified by age at death

	All births		Age at death 2–11 months										Age at death 1–4 years									
	n=761 172		RTI					SUD					RTI					SUD				
			n=98					n=228					n=112					n=54				
			Chronic condition		No chronic condition			Chronic condition		No chronic condition			Chronic condition		No chronic condition			Chronic condition		No chronic condition		
			n=80		n=18			n=35		n=193			n=93		n=19			n=14		n=40		
	n	%	n	%	n	%	P values*	n	%	n	%	P values	n	%	n	%	P values	n	%	n	%	P values
Birth characteristics																						
Sex																						
Male	392 358	51.55	47	58.75	13	72.22	0.423	16	45.71	115	59.59	0.140	49	52.69	10	52.63	1.000	6	42.86	27	67.50	0.123
Female	368 814	48.45	33	41.25	5	27.78		19	54.29	78	40.41		44	47.31	9	47.37		8	57.14	13	32.50	
Preterm birth (<37 weeks)	41 149	5.41	25	31.25	<5†		0.385	7	20.00	34	17.62	0.943	24	25.81	<5		0.031	<5		6	15.00	*0.086*
Missing	36 270	4.77	9	11.25	<5			<5		12	6.22		9	9.68	0	0.00		<5		<5		
Socioeconomic																						
Teenage mother (<20 years)	52 748	6.93	10	12.50	<5		0.575	<5		35	18.13	0.169	6	6.45	<5		0.417	<5		6	15.00	0.372
Missing	54 732	7.19	6	7.50	<5			<5		31	16.06		8	8.60	0	0.00		<5		<5		
Most+second most deprived quintile	336 907	44.26	41	51.25	10	55.56	0.946	22	62.86	108	55.96	0.760	51	54.84	7	36.84	0.345	11	78.57	19	47.50	0.345
Health																						
Had missed or delayed infant vaccination	52 838	6.94	NA					NA					19	20.43	<5		0.188	<5		<5		0.358
Death																						
Had a postmortem			24	30.00	17	88.89	<0.001	32	91.43	187	96.89	0.145	24	25.81	17	89.47	<0.001	12	85.71	33	82.50	1.000

*Chronic condition versus no chronic condition.

†Statistical disclosure control for Scottish administrative data requires disclosure protection for cell sizes smaller than 5.

NA, not applicable; RTI, respiratory tract infection; SUD, sudden unexplained death.

## Results

**Figure 1:**
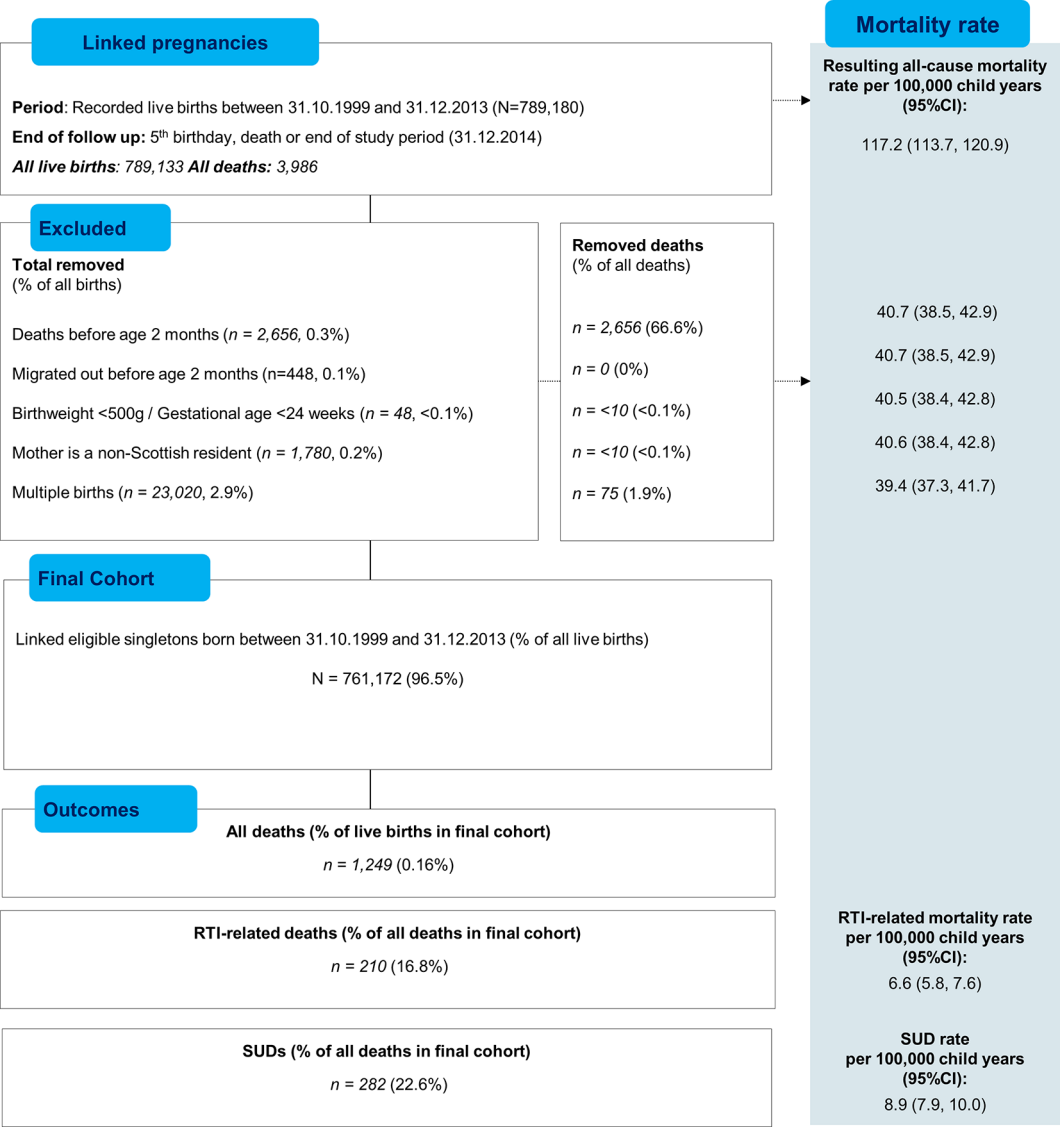
Selection of cohort and denominators. RTI, respiratory tract infection; SUD, sudden unexplained death.

The study population comprised 761 172 eligible singletons aged 2 months to 4 completed years and included 3 292 731.29 child-years of follow-up (mean 4.32 years, SD 1.19). The derivation of the cohort is shown in figure 1. A chronic condition was recorded in hospital admission records between birth and 5 years of age or on the death certificate for 1 in 10 children (n=72 901).

Of all deaths (n=1249) between 2 months and 4 years of age, 39.4% were accounted for by RTI-related causes or SUD (table 1). Underlying chronic conditions were recorded in 82.4% of RTI deaths (n=173) and 17.4% (n=49) of SUD deaths. Only 28% of children who died with RTI and an underlying chronic condition had a postmortem compared with 90% of children who died without chronic conditions (table 1).

Among children surviving to 2 months, 7 in 100 000 children died with RTI between 2 months and 4 years and 9 in 100 000 died of SUD.

Children with chronic conditions and those born preterm had higher RTI mortality and SUD rates compared with children without these characteristics (table 2). Children born to teenage mothers had higher SUD rates than children born to mothers aged 20 or more years but not higher RTI mortality rates.

**Table 2:** Crude mortality rates by risk factor according to type of death and age at death (per 100 000 child-years with 95% CIs)

	2–11 completed months		1–4 completed years	
	RTI-related death n=98	SUD n=228	RTI-related death n=112	SUD n=54
No recorded chronic condition	3.01 (1.89, 4.77)	32.23 (27.99, 37.11)	0.83 (0.53, 1.31)	1.76 (1.29, 2.40)
Recorded chronic condition	251.35 (201.89, 312.93)	109.97 (78.96, 153.16)	35.60 (29.06, 43.63)	5.36 (3.17, 9.04)
No teenage mother (>19 years)	14.22 (11.37, 17.77)	28.80 (24.62, 33.70)	4.43 (3.63, 5.41)	1.89 (1.39, 2.57)
Teenage mother (<20 years)	32.00 (18.95, 54.02)	86.85 (63.20, 119.36)	4.32 (2.16, 8.63)	5.40 (2.90, 10.03)
Not preterm (>36 weeks)	11.73 (9.28, 14.83)	31.34 (27.16, 36.17)	3.63 (2.94, 4.47)	1.83 (1.36, 2.46)
Preterm (<37 weeks)	82.24 (56.79, 119.12)	120.44 (88.68, 163.60)	18.10 (12.23, 26.79)	7.24 (3.90, 13.46)

RTI, respiratory tract infection; SUD, sudden unexplained death.

### Multivariable analyses

We report the multivariable analysis by age group. The proportional hazards assumption of the Cox models was met.

#### Ages 2–11 months

**Figure 2:**
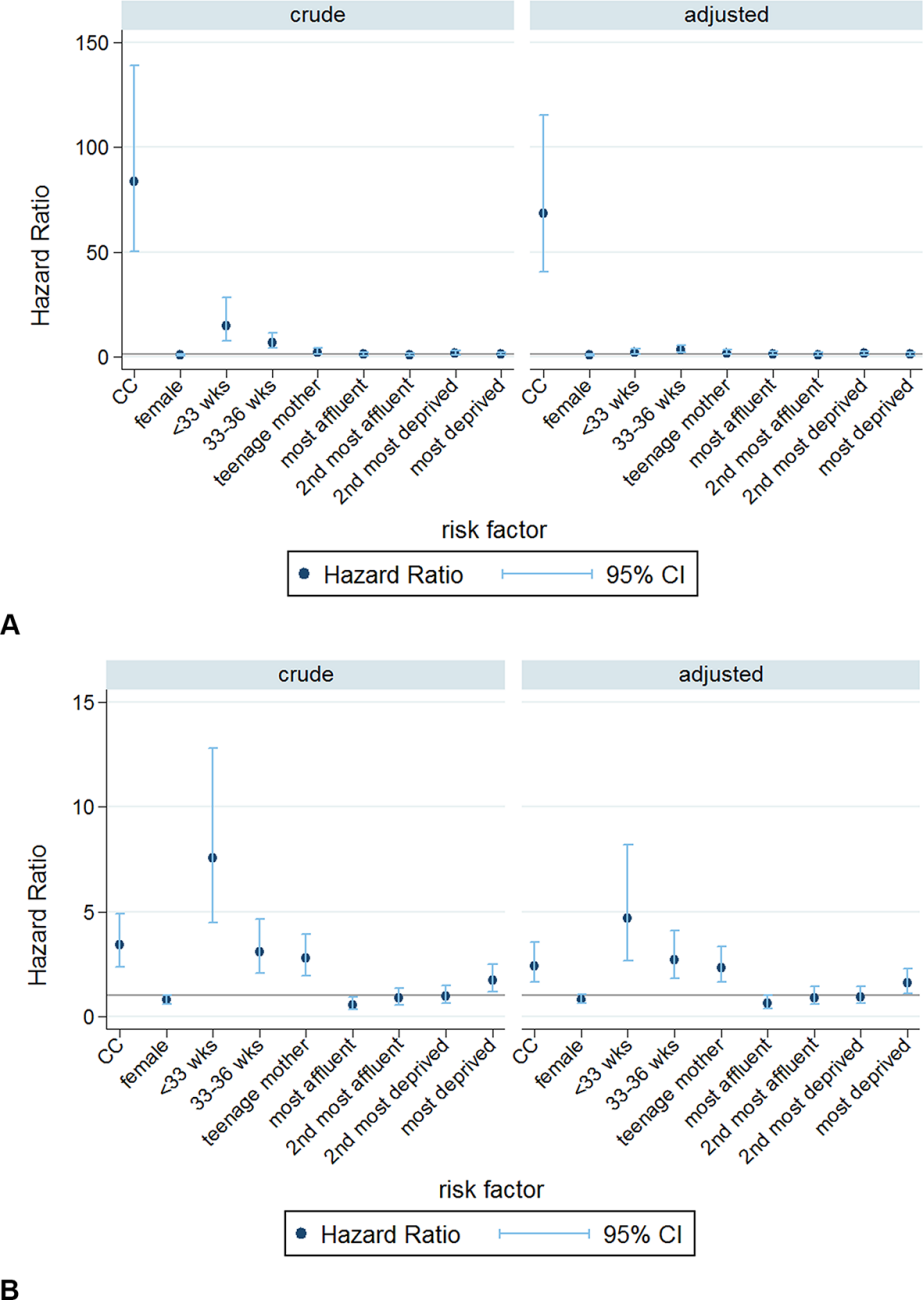
Crude and adjusted Cox HRs for (A) respiratory tract infection (RTI)-related mortality and (B) sudden unexplained death (SUD), ages 2–11 months. CC, chronic conditions.

RTI-related deaths and SUDs together accounted for 47.5% of all deaths in children aged 2–11 months. Presence of a chronic condition was strongly associated with increased mortality related to RTI or SUD (figure 2A,B and online supplementary table 1a,b). Presence of a chronic condition increased the risk of RTI-related death 69-fold, and of SUD twofold, after adjusting for other risk factors.

Preterm birth increased risks of both outcomes and attenuated the effects of chronic conditions. Having a teenage mother was associated with SUD and RTI-related mortality and area-level deprivation was associated only with SUD. Inclusion of socioeconomic risk factors did not appreciably reduce the associations between presence of an underlying chronic condition and RTI mortality or SUD (figure 2A,B and online supplementary table 1a,b).

#### Ages 1–4 years

**Figure 3:**
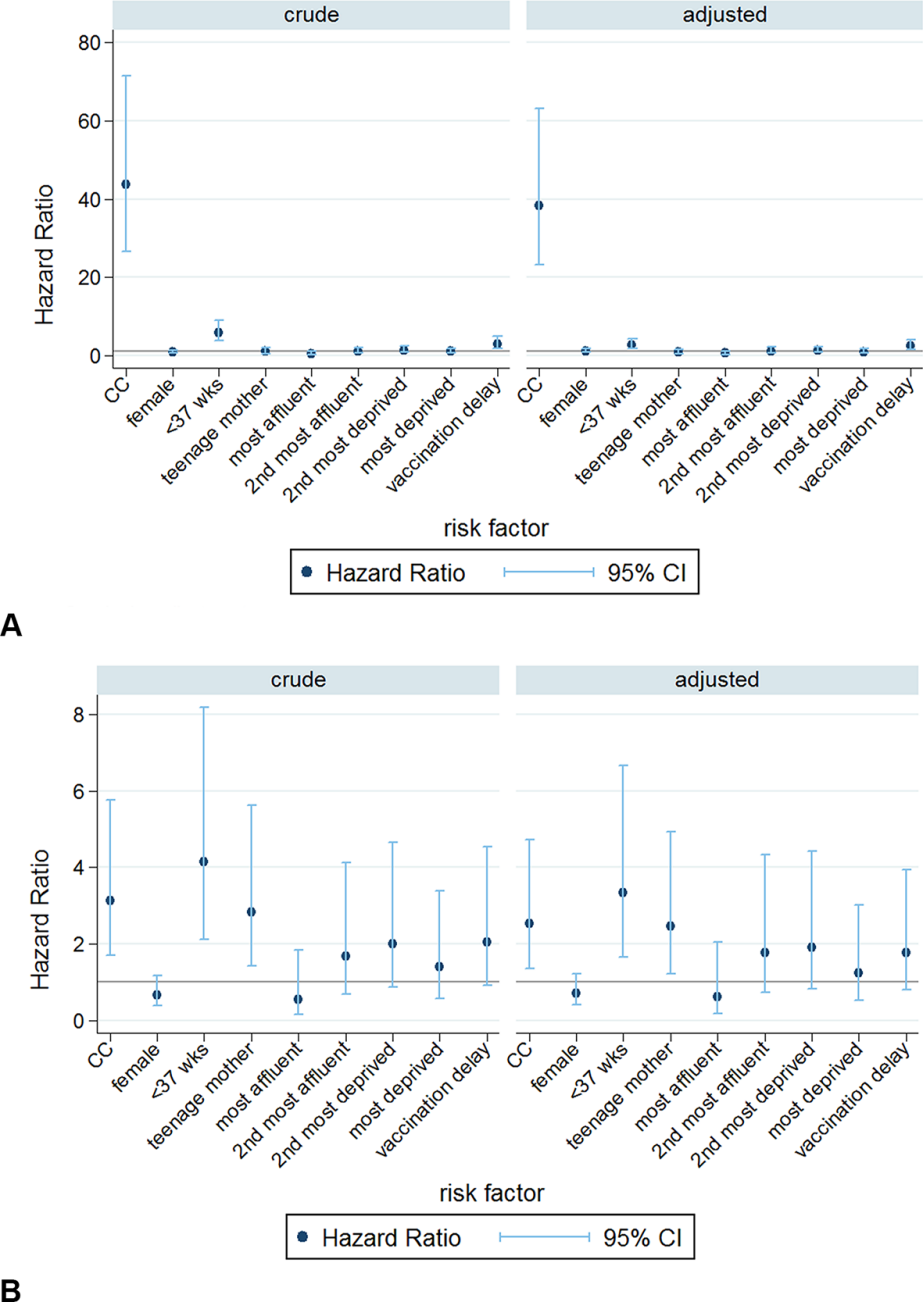
Crude and adjusted Cox HRs for (A) respiratory tract infection (RTI)-related mortality and (B) sudden unexplained death (SUD), ages 1–4 completed years.

Altogether, RTI-related deaths and SUDs accounted for 32.2% of all deaths in 1–4 year-olds. Presence of a chronic condition increased the risk of RTI-related death 38-fold, and of SUD threefold, after adjustment for other risk factors (figure 3A,B and online supplementary table 2a,b)

Preterm birth increased the risk of both outcomes but was strongest for SUD. The increased risk of SUD among children with a teenage mother persisted among 1–4 year-olds. After adjustment for other risk factors, area-level deprivation was not associated with RTI-related death or SUD. Delayed infant vaccination was associated with RTI-related mortality but not SUD. Sex was not associated with mortality related to RTI or SUD in either age group.

## Discussion

In Scotland, 7 in 100 000 children died with RTI aged 2 months to 4 years and 9 in 100 000 children died of SUD. In children ever hospitalised with a chronic condition, the rates of RTI-related death and SUD between 2 months and 4 years were 54 and 15 per 100 000 children per year, respectively. For RTI death, presence of chronic conditions was the strongest measured risk factor, whereas for SUD it was gestational age.

### Strengths and limitations

This is the first cohort study to investigate the role of chronic conditions in RTI-related deaths and SUDs for a whole country. Linkage of longitudinal hospitalisation, vaccination, outmigration and death records for all children born in Scotland made it possible to determine the contribution of chronic conditions to two major causes of death under age 5 and avoided selection bias due to referral or migration. Although we used whole-country data, since deaths in children are rare, the statistical power was too low to present findings disaggregated by types of chronic conditions.

Contributory causes of death are not systematically recorded in the death registration which could have diluted the association between RTIs and chronic conditions. We minimised underascertainment of RTI-related deaths by classifying a death as RTI related if RTI-related ICD-10 codes were recorded in hospitalisation records within 30 days prior to a non-injury death. This method has been applied in a previous study.^9^

We likely underascertained chronic conditions that are mostly managed in primary care, such as wheeze or asthma. Our results are therefore generalisable only to chronic conditions severe enough to require hospital admission or to be recorded on the death certificate.

Our findings for children with chronic conditions in Scotland are likely to be strongly influenced by practices in the two main specialist paediatric intensive care centres in Glasgow and Edinburgh and may not be generalisable to the whole of the UK.

**Figure 4:**
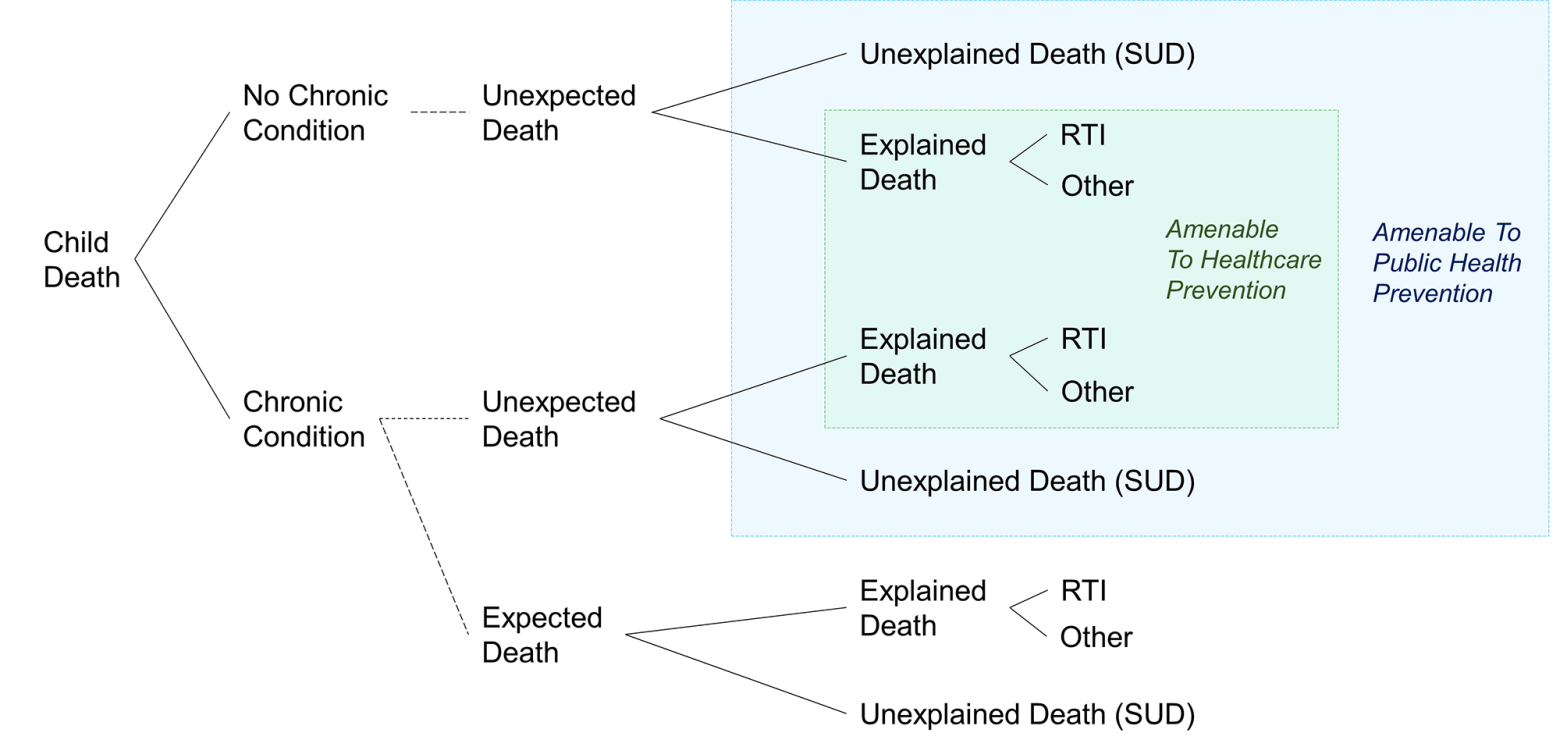
Proposed mortality surveillance of public health and healthcare amenable deaths by categorising children with an underlying chronic condition or not, ascertained from hospital or death records and whether death was recorded as ’expected on death' registration and hospital records. Dashed Iines represent what administrative data-based research currently cannot distinguish. RTI, respiratory tract infection-related death; SUD, sudden unexplained death.

SUD in children with a chronic condition is also likely to be under-registered, due to uncertainty about whether death is an expected complication of the underlying condition. In this case, clinicians may *assume* that a sudden death was caused by the chronic condition as illustrated in figure 4. Such cases would not have been included in our analyses, and we would have underestimated the rate of SUD and its association with chronic conditions.

A further weakness is the meaning of delayed vaccination status. While delayed vaccination could indicate lack of uptake of preventive health services, it could also indicate poor recording, vaccination given in hospital or vaccination deferred due to illnesses.

In this study, RTI-related death and SUD were analysed as mutually exclusive outcomes, but they are likely to share common social and medical causal pathways. RTI-related death and SUD lie on a spectrum of social and medical need, with chronic conditions present as a major, clinically identifiable risk factor in both groups. SUD occurred more frequently than RTI-related death in previously healthy children, but we found an independent association with socioeconomic factors, even after accounting for preterm birth and chronic conditions. In contrast, RTI-related death occurred predominantly in children with chronic conditions and there was no evidence of a statistically significant association with teenage pregnancy, nor an apparent linear association with area-level deprivation after accounting for birth characteristics and chronic conditions. Since we analysed these outcomes as mutually exclusive, we potentially underestimated RTI-related deaths because deaths which met both conditions were classified as SUD. Our findings of increased risks of RTI-related death and SUD associated with chronic conditions, preterm birth and, in case of SUD, area-level deprivation and young maternal age are consistent with previous studies.^2 5 8 10 25–28^

### Implications

More than 80% of RTI-related deaths occurred in children with underlying chronic conditions. The finding that only one-quarter of these children had a record indicating postmortem suggests that many of these deaths were expected, possibly because RTI was part of the terminal process. We recommend adding whether death was expected to the death registration and/or administrative hospital databases to improve identification of deaths that might be avoidable through prevention by healthcare intervention.

Seventeen per cent of SUDs were in children with a record indicating a chronic condition. SUD is amenable to effective public health interventions such as efforts to increase safe infant sleeping and reduce parental alcohol, smoking and drug use.^29–31^ The strong association between chronic conditions and SUD, independent of social risk factors, suggests that these universal preventive strategies are likely to be of particular benefit for children with chronic conditions.

Our findings demonstrate that the rate of unexpected and potentially avoidable child mortality remains unknown (blue and green boxes, figure 4). Unexpected deaths that are explained by conditions that are preventable or treatable, such as RTI, could be avoidable (green shading, figure 4). However, without the distinction of expected or not, RTI-related deaths are not a useful indicator of avoidable mortality as it is not possible to quantify the proportion of deaths that are expected. SUD is a useful indication of potentially avoidable mortality but likely underestimates the true number of unexpected deaths as sudden death in children with chronic conditions may be attributed to the condition itself. Improved estimation of potentially avoidable child mortality through public health measures or healthcare could be achieved by recording whether death was expected on death registration and hospital records.

## Conclusions

Children with chronic conditions were at substantially increased risk of RTI-related death and of SUD. While SUD remains a useful indicator of avoidable mortality, the vast majority of RTI deaths were in children with chronic conditions. Some of these are likely to have been expected deaths. The introduction of an indicator of whether death was expected would allow improved estimation of potentially avoidable child mortality.

10.1136/archdischild-2017-314098.supp2 Supplementary file 2

10.1136/archdischild-2017-314098.supp3 Supplementary file 3

10.1136/archdischild-2017-314098.supp4 Supplementary file 4

10.1136/archdischild-2017-314098.supp5 Supplementary file 5
