# Supplementary material for: Avoidable mortality from respiratory tract infection and sudden unexplained death in children with chronic conditions: a data linkage study
Source: Arch Dis Child. 2018 Jul 14;103(12):1125–31. doi: 10.1136/archdischild-2017-314098 (PMC6287561; doi:10.1136/archdischild-2017-314098)
Supplement: Supplementary file 1 [file archdischild-2017-314098supp001.pdf]

Supplementary Table 3

International Classification of Diseases version 10 codes used to identify children with chronic conditions

Type of chronic condition	Categories	Codes
Mental health/behavioural	Substance abuse	E24.4, F10-F19, F55, G24.0, G31.2, G40.5, G62.1, G72.0, G72.1, I42.6, K29.2, K70, K85.2, K85.3, K86.0, O35.4, R78.1-R78.5, Y47, Y49, Z50.2, Z50.3, Z71.4, Z71.5, Z72.2, Z86.4
	Self-harm	X60-X84, Y10-Y34, Y87.0, Y87.2, Z91.5
	Other mental health problems	F00-F01, F02.8, F03-F09, F20-F48, F50, F53, F54, F59, F60-F69, F99, Z09.3, Z50.4, Z86.5, Z91.4
	Behavioural/developmental disorders	F70-F79, F80.0-F80.2, F80.8, F80.9, F81-F84, F88, F89, F90-F98
Cancer/blood disorders	Neoplasms	C00-C97, D00-D02, D05-D09, D12, D13, D14.1-D14.4, D15, D20, D32-D35, D37-D48, D63.0, E34.0, E88.3, G13.0, G13.1, G53.3, G55.0, G63.1, G73.1, G73.2, G94.1, M36.0, M36.1, M49.5, M82.0, M90.6, M90.7, N08.1, N16.1, Y43.1-Y43.3, Y84.2, Z08, Z51.0-Z51.2, Z54.1, Z54.2, Z85, Z86.0, Z92.3
	Immunological disorders	D80-D84, G53.2, Q98.0
	Anaemia and other blood disorders	D50, D56.0-D56.2, D56.4, D56.8, D56.9, D57.0-D57.2, D57.8, D58, D61.0, D61.9, D64, D66, D67, D68.0-D68.2, D68.4-D68.9, D69, D70-D76, M36.2-M36.4, M90.4, N08.2, Z86.2
Chronic infections	HIV	B20-B24, F02.4, R75, Z21
	Tuberculosis	A15-A19, E35.0, K23.0, K67.3, K93.0, M01.1, M49.0, P37.0
	Other	A50, A81, B18, B37.1, B37.5, B37.6, B37.7, B38.1, B39.1, B40.1, B44.0, B44.7, B45, B46, B48.7, B50.0, B50.8, B51.0, B51.8, B52.8, B52.0, B55, B57.2-B57.5, B58.0, B59, B67, B69, B73, B74, B78.7, B90-B94, F02.1, K23.1, K93.1, M00, N33.0, P35.0-P35.2, P35.8, P35.9, P37.1
Respiratory	Asthma and chronic lower respiratory disease	J41-J47
	Cystic fibrosis	E84, P75
	Injuries	S17, S27, S28, T27, T91.4
	Congenital anomalies	Q30-Q37, Q79.0
	Other	G47.3, J60-J70, J80-J86, J96.1, J98, P27, Y55.6, Z43.0, Z93.0, Z94.2
Metabolic/endocrine/digestive/renal/ genitourinary	Diabetes	E10-E14, G59.0, G63.2, I79.2, M14.2, N08.3, O24, Y42.3
	Other endocrine	E00, E03.0, E03.1, E07.1, E22.0, E23.0, E25, E26.8, E29.1, E31, E34.1, E34.2, E34.5, E34.8, G13.2, G73.5, Y42.1
	Metabolic	D55, E70-E72, E74-E78, E79.1-E79.9,E80.0-E80.3, E80.5, E80.7, E83, E85, E88.0, E88.1, E88.2, E88.8, E88.9, G73.6, L99.0, M14.4, M14.3, N16.3
	Digestive	K20, K21.0, K22, K23.8, K25-K28, K29.0, K29.1, K29.3-K29.9, K31, K50-K52, K55, K57, K59.2, K63.0-K63.3, K66, K72-K76, K80-K83, K85.0, K85.1, K85.8, K85.9, K86.1-K86.9, K87.0, K90, M07.4, M07.5, M09.1, M09.2, T86.4, Z43.2-Z43.4, Z46.5, Z90.3, Z90.4, Z93.2-Z93.5
	Renal/GU	D63.8, G63.8, G99.8, I68.8, M90.8, N08.4, N00-N05, N07, N11-N15, N16.0, N16.2, N16.4, N16.5, N16.8, N18, N19, N20-N23, N25, N26, N28, N29, N31, N32, N33.8, N35, N36, N39.1, N39.3, N39.4,

		N40-N42, N70-N74, N80-N82, N85, N86, N87,N88, P96.0, T82.4, T83.1, T83.2, T83.4-T83.9, T85.5, T86.1, Y60.2, Y61.2, Y62.2, Y84.1, Z49, Z93.6, Z94.0, Z99.2
	Congenital anomalies of the digestive/renal/GU system	Q38.0, Q38.3, Q38.4, Q38.6-Q38.8, Q39, Q40.2, Q40.3, Q40.8, Q40.9, Q41, Q42, Q43.1, Q43.3-Q43.7, Q43.9, Q44, Q45, Q50.0, Q51, Q52.0-Q52.2, Q52.4, Q54.0-Q54.3, Q54.8, Q54.9, Q55.0, Q55.5, Q56, Q60.1, Q60.2, Q60.4-Q60.6, Q61, Q62.0-Q62.6, Q62.8, Q63.0-Q63.2, Q63.8, Q63.9, Q64, Q79.2-Q79.5, Q87.8, Q89.1, Q89.2
	Injuries	S36, S37, S38, S39.6, S39.7, T06.5, T28, T91.5
	Other/unspecific	E66, G63.3, G99.0, M14.5, N92, Z86.3, Z93.8
Musculoskeletal/skin	Musculoskeletal/connective tissue	G55.1-G55.3, G63.5, G63.6, G73.7, J99.0, J99.1, L62.0, M05, M06, M07.0-M07.3, M07.6, M08, M09.8, M10-M13, M14.0, M14.6, M14.8, M30-M35, M40-M43, M45-M48,M50-M54, M60-M62, M63.8, M80.1-M80.9, M81.1-M81.9, M82.1, M82.8, M84.0-M84.2, M84.8, M84.9, M85, M86.3-M86.6, M89, M90.0, M91-M94, N08.5, Y45.4
	Skeletal injuries/amputations	S13, S22.0-S22.2, S22.5, S23, S32, S33, S68.3, S68.4, S68.8, S77, S78, S87, S88, S97, S98.0, S98.2-S98.4, T02, T04, T05, T20.3, T20.7, T21.3, T21.7, T22.3, T22.7, T23.2, T23.3, T23.6, T23.7, T24.3, T24.7, T25.2, T25.3, T25.6, T25.7, T29.3, T29.7, T30.3, T30.7, T31.2-T31.9, T32.2-T32.9, T87.3-T87.6, T91.2 T91.8, T92.6, T93.1, T93.4, T93.6, T94.0, T94.1, T95.0, T95.1, T95.4, T95.8, T95.9, Y83.5, Z89.1, Z89.2, Z89.5-Z89.8, Z97.1
	Chronic skin disorders	L10, L11.0, L11.8, L11.9, L12-L14, L28, L40-L45, L57, L58.1, L59, L87, L88, L90, L92, L95, L93, L98.5, M09.0, Q80, Q81, Q87.0-Q87.5, Q89.4
	Congenital anomalies	Q18.8, Q65.0-Q65.2, Q65.8, Q65.9, Q67.5, Q68.2, Q68.3-Q68.5, Q71-Q73, Q74, Q75.3-Q75.9, Q76.1-Q76.4, Q77, Q78, Q79.6, Q79.8, Q82.0-Q82.4, Q82.9, Q86.2, Q89.7-Q89.9
Neurological	Epilepsy	F80.3, G40.0-G40.4, G40.6-G40.9, G41, R56.8, Y46.0-Y46.6
	Cerebral palsy	G80-G83
	Injuries of brain, nerves, eyes or ears	S05-S08, S12, S14, S24, S34, S44, S54, S64, S74, S84, S94, T06.0-T06.2, T26, T90.4, T90.5, T91.1, T91.3, T92.4,
	Chronic eye conditions	H05.1-H05.9, H13.3, H17, H18, H19.3, H19.8, H21, H26, H27, H28.0-H28.2, H31, H32.8, H33, H34, H35, H40, H42.0, H43, H44, H47, H54.0- H54.2, H54.4, T85.2, T85.3, Z44.2
	Chronic ear conditions	H60.2, H65.2-H65.4, H66.1-H66.3, H69.0, H70.1, H73.1, H74.0-H74.3, H75.0, H80, H81.0, H81.4, H83.0, H83.2, H90.0, H90.3, H90.5, H90.6, H91, Z45.3
	Perinatal conditions	P10, P21.0, P52, P57, P90, P91.1, P91.2, P91.6
	Congenital anomalies of neurological or sensory systems	Q00-Q07, Q10.4, Q10.7, Q11-Q12, Q13.0-Q13.4, Q13.8, Q13.9, Q14-Q16, Q75.0, Q75.1, Q85, Q86.0, Q86.1, Q86.8, Q90-Q93, Q95.2, Q95.3, Q97, Q99
	Other	F02.2, F02.3,G00-G09, G10-G12, G13.8, G14, G20-G23, G24.1-G24.9, G25-G30, G31.0-G31.1, G31.8, G31.9, G32-G37, G43-G46, G47.0-G47.2, G47.4-G47.9, G50-G52, G53.0, G53.1, G53.8, G54, G55.8, G56-G58, G59.8, G60, G61, G62.0, G62.2-G62.9, G64, G70, G71,G72.2-G72.9, G73.0, G73.3, G90-G93, G94.2, G94.8, G95, G96, G98, G99.1,

		G99.2, I60-I67, I68.0, I68.2, I69, I72.0, I72.5, T85.0, T85.1, Y46.7-Y46.8, Z98.2
Cardiovascular	Congenital heart disease	Q20-Q26, Q89.3
	Other	I00-I28, I31-I39, I41, I42.0-I42.5, I42.7-I42.9, I43.0, I43.1, I43.2-I43.8, I44.1-I44.7, I45.1-I45.9, I46-I51, I52.8, I70-I71, I72.1-I72.4, I72.8, I72.9, I73-I77, I79.0, I79.1, I79.8, I81-I82, I98-I99, M03.6, N08.8, Q27, Q28, S26, T82.0-T82.3, T82.5-T82.9, T86.2, Y60.5, Y61.5, Y62.5, Y84.0, Z45.0, Z50.0, Z94.1, Z95
Codes indicating non-specific chronic condition	-	R62, R63.3, Z43.1, Z51.5, Z75.5, Z93.1, Z99.3
