# Supplementary material for: Avoidable mortality from respiratory tract infection and sudden unexplained death in children with chronic conditions: a data linkage study
Source: Arch Dis Child. 2018 Jul 14;103(12):1125–31. doi: 10.1136/archdischild-2017-314098 (PMC6287561; doi:10.1136/archdischild-2017-314098)
Supplement: Supplementary file 2 [file archdischild-2017-314098supp002.pdf]

Supplementary Table 1 (a)

Risk factors associated with RTI related mortality for children aged 2 months-11 months in Scotland 2000 – 2014, Multivariate Cox regression, 15 multiple imputations

Risk Factors*	RTI deaths / 100,000 child years N=98	Hazard Ratio (95% CI)					
		Model 1		Model 2[a]		Model 3[b]	
		Chronic conditions		Birth characteristics		Socio-economic	
**Chronic condition up to age 1y**	80 / 0.32	83.54	(50.10, 139.30)	69.54	(42.20, 117.38)	68.48	(40.57, 115.60)
**Female**	38 / 3.06			0.81	(0.54, 1.22)	0.81	(0.54, 1.21)
**Gestational age (weeks)**							
<33	n/a			2.03	(1.03, 4.00)	2.00	(1.02, 3.95)
33-36				3.28	(1.93, 5.57)	3.24	(1.91, 5.52)
37+				base		base	
**Teenage pregnancy (<20y)**	n/a					1.81	(1.02, 3.23)
**Deprivation quintile**							
1 (most deprived)	23 / 1.46					1.05	(0.56, 1.97)
2	28 / 1.33					1.47	(0.80, 2.68)
3	17 / 1.26					base	
4	13 / 1.12					0.92	(0.45, 1.89)
5 (least deprived)	17 / 1.14					1.20	(0.61, 2.35)

a: adjusted for chronic conditions, sex and gestational age.

b: adjusted for chronic conditions, sex, gestational age and SES

n/a: not applicable for imputed variables since values vary by imputation

* according to our definition vaccine uptake is not estimable for children aged <1 year
