# Supplementary material for: Avoidable mortality from respiratory tract infection and sudden unexplained death in children with chronic conditions: a data linkage study
Source: Arch Dis Child. 2018 Jul 14;103(12):1125–31. doi: 10.1136/archdischild-2017-314098 (PMC6287561; doi:10.1136/archdischild-2017-314098)
Supplement: Supplementary file 3 [file archdischild-2017-314098supp003.pdf]

Supplementary Table 1 (b)
Risk factors associated with SUD for children aged 2 months-11 months in Scotland 2000 – 2014, Multivariate Cox regression, 15 multiple imputations

Risk Factors*	SUD deaths / 100,000 child years N=228	Hazard Ratio (95% CI)		
		Model 1	Model 2[a]	Model 3[b]
		Chronic conditions	Birth characteristics	Socio-economic
**Chronic condition up to age 1y**	49 / 3.21	3.40 (2.37, 4.89)	2.46 (1.67, 3.63)	2.42 (1.67, 3.63)
**Female**	97 / 3.06		0.81 (0.63, 1.06)	0.82 (0.63, 1.02)
**Gestational age (weeks)**	n/a			
<33			5.01 (2.84, 8.82)	4.67 (2.77, 7.77)
33-36			2.83 (1.88, 4.27)	2.71 (1.96, 4.06)
37+			base	Base
**Teenage pregnancy (<20y)**	n/a			2.33 (1.75, 3.32)
**Deprivation quintile**				
1 (most deprived)	86 / 1.46			1.59 (1.10, 2.29)
2	44 / 1.33			0.94 (0.61, 1.42)
3	43 / 1.26			base
4	33 / 1.12			0.90 (0.57, 1.41)
5 (least deprived)	22 / 1.14			0.61 (0.36, 1.02)

a: adjusted for chronic conditions, sex and gestational age.
b: adjusted for chronic conditions, sex, gestational age and SES
n/a: not applicable for imputed variables since values vary by imputation
* according to our definition vaccine uptake is not estimable for children aged <1 year
