# Supplementary material for: Avoidable mortality from respiratory tract infection and sudden unexplained death in children with chronic conditions: a data linkage study
Source: Arch Dis Child. 2018 Jul 14;103(12):1125–31. doi: 10.1136/archdischild-2017-314098 (PMC6287561; doi:10.1136/archdischild-2017-314098)
Supplement: Supplementary file 4 [file archdischild-2017-314098supp004.pdf]

Supplementary Table 2 (a)

Risk factors associated with RTI for children aged 1-4 completed years in Scotland 2000 – 2014, Multivariate Cox regression, 15 multiple imputations

Risk Factors	RTI deaths / 100,000 child years N=112	Hazard Ratio (95% CI)							
		Model 1		Model 2[a]		Model 3[b]		Model 4[c]	
		Chronic conditions		*Birth characteristics*		*Socio-economic*		*Health services contact*	
**Chronic condition up to age 5y**	93 / 2.61	43.65	(26.65, 71.50)	38.91	(23.62, 64.09)	38.83	(23.57, 63.98)	38.32	(23.26, 63.14)
**Female**	53 / 12.35			1.16	(0.80, 1.68)	1.16	(0.80, 1.68)	1.16	(0.80, 1.68)
**Gestational age (weeks)**									
<37	n/a			2.86	(1.86, 4.40)	2.82	(1.83, 4.34)	2.74	(1.78, 4.22)
37+				base		base			
**Teenage pregnancy (<20y)**	n/a					0.79	(0.38, 1.64)	0.77	(0.37, 1.58)
**Deprivation quintile**									
1 (most deprived)	26 / 5.86					0.97	(0.55, 1.72)	0.94	(0.53, 1.66)
2	32 / 5.36					1.33	(0.77, 2.29)	1.32	(0.77, 2.27)
3	22 / 5.06					base		base	
4	22 / 4.50					1.17	(0.65, 2.12)	1.18	(0.66, 2.14)
5 (least deprived)	10 / 4.59					0.53	(0.25, 1.13)	0.55	(0.26, 1.15)
**Vaccination delay in infancy**	20 / 1.75							2.47	(1.52, 4.01)

a: adjusted for chronic conditions, sex and gestational age.

b: adjusted for chronic conditions, sex, gestational age and SES

c: adjusted for chronic conditions, sex, gestational age, SES and health services contact

n/a: not applicable for imputed variables since values vary for each imputation
