# Supplementary material for: Avoidable mortality from respiratory tract infection and sudden unexplained death in children with chronic conditions: a data linkage study
Source: Arch Dis Child. 2018 Jul 14;103(12):1125–31. doi: 10.1136/archdischild-2017-314098 (PMC6287561; doi:10.1136/archdischild-2017-314098)
Supplement: Supplementary file 5 [file archdischild-2017-314098supp005.pdf]

Supplementary Table 2 (b)
Risk factors associated with SUD related mortality for children aged 1-4 completed years in Scotland 2000 – 2014, Multivariate Cox regression, 15 multiple imputations

Risk Factors	SUD deaths / 100,000 child years<br>N=54	Hazard Ratio (95% CI)							
		Model 1		Model 2[a]		Model 3[b]		Model 4[c]	
		Chronic conditions		*Birth characteristics*		*Socio-economic*		*Health services contact*	
**Chronic condition up to age 5y**	14 / 2.61	3.13	(1.70, 5.76)	2.61	(1.40, 4.87)	2.55	(1.37, 4.75)	2.53	(1.36, 4.71)
**Female**	21 / 12.35			0.71	(0.41, 1.23)	0.71	(0.41, 1.22)	0.71	(0.41, 1.23)
**Gestational age (weeks)**									
< 37	n/a			3.48	(1.74, 6.96)	3.38	(1.69, 6.75)	3.33	(1.67, 6.66)
37+				base		base		base	
**Teenage pregnancy (<20y)**	n/a					2.50	(1.25, 5.02)	2.45	(1.22, 4.93)
**Deprivation quintile**									
1 (most deprived)	13 / 5.86					1.28	(0.53, 3.09)	1.25	(0.52, 3.03)
2	17 / 5.36					1.93	(0.83, 4.46)	1.91	(0.82, 4.43)
3	8 / 5.06					base		base	
4	12 / 4.50					1.77	(0.72, 4.32)	1.77	(0.72, 4.34)
5 (least deprived)	4 / 4.59					0.61	(0.18, 2.02)	0.61	(0.18, 2.05)
**Vaccination delay in infancy**	7 / 1.75							1.77	(0.80, 3.93)

a: adjusted for chronic conditions, sex and gestational age.

b: adjusted for chronic conditions, sex, gestational age and SES

c: adjusted for chronic conditions, sex, gestational age, SES and health services contact

n/a: not applicable for imputed variables since values vary for each imputation
